# Supplementary material for: MET in Non-Small-Cell Lung Cancer (NSCLC): Cross ‘a Long and Winding Road’ Looking for a Target
Source: Cancers (Basel). 2023 Sep 28;15(19):4779. doi: 10.3390/cancers15194779 (PMC10571577; doi:10.3390/cancers15194779)
Supplement: Supplementary file 1 [file cancers-15-04779-s001.zip › cancers-2580971-supplementary.pdf]

Table S1 The randomized phase III trials of onartuzumab and tivantinib

Trial Reference	Key El. Criteria	Pt N	Drugs	mPFS HR (95% CI)	Principal endpoint OS Median HR (95% CI)	Comments
METLung Spigel 2017 [53]	MET +*	250 vs 249	Onartuzumab + E Vs Placebo + E	2.7 months Vs 2.6 months 0.99 (0.81-1.20)	6.8 months Vs 9.1 months 1.27 (0.98-1.65)	No Predictive factors
Scagliotti 2015 [54]	Non-Sq	526 Vs 522	Tivantinib + E Vs Placebo + E	3.6 months Vs 1.9 months 0.74 (0.62- 0.89	8.5 months Vs 7.8 months 0.98 (0.84-1.15	Trend for OS for MET OE

*MET + stands for ≥ 50% of tumour cells with IHC scores of 2+ (moderate) or 3+ (strong) levels of MET

Abbreviations: El.= eligibility; Pt N = patient number; PFS = progression-free survival; OS = overall survival; HR = hazard ratio; Non-sq = non-squamous carcinoma; E = erlotinib; MET OE = MET overexpression

Table S2- Summary of cases report of crizotinib in patients with NSCLC harbouring MET alterations

Reference	Age	Sex	Smok. S	Hist.	MET Alteration	Line	Response	F-UP
Ou 2011 [58]	77	F	Former	ADK	MET/CEP7 ratio >5.0	2nd	PR	74 mos°
Schwab 2014 [59]	73	M	NR	Sq	MET/CEP7 ratio >2.2	1st	CR	3 mos*
Caparica 2017 [60]	56	M	Current	ADK	MET/CEP7 ratio 9.94	2nd	PR	8 mos
Jenkins 2015 [61]	86	M	Never	ADK	METex14 c. 2887-18_2887-7del12	2nd	PR	2 mos§
Waqar 2015 [62]	71	M	Former	ADK	METex14 p.D1028H	2nd	PR	6 mos
Paik 2015 [63]	80	M	Former	ADK	METex14 c.3024_3028del	3rd	PR	3.6 mos
Paik 2015 [63]	65	M	Current	ADK	METex14 c3024_3028delAGAA	3rd	PR	4.6 mos
Paik 2015 [63]	90	F	Never	ADK	METex14 p.V1001_F1007 del	3rd	PR	3.1 mos
Awad 2016 [36]	64	F	Never	ADK	METex14ᶝ c.3028G.A	2nd	PR	8 mos
Davies 2017 [64]	74	F	Never	ADK	HLA-DRB1-MET	2nd	CR	8 mos
Wang 2019 [65]	67	F	Never	ADK	METex14 3077_3082 + 9del	4th	PR	12 mos
Wang 2019 [65]	77	F	Former	ADK	METex14 3082G > C	3rd	PR	6 mos
Wang 2019 [65]	62	M	Never	ADK	METex14 NR	5th	PR	4 mos
Wang 2019 [65]	73	M	Former	Sq	METex14 NR	2nd	PD	1 mo
Wang	78	M	Never	ADK	METex14	3rd	NE	1 mo*

2019 [65]					2942-4_2942-43			
Wang 2019 [65]	56	F	Former	ADK	METex14 skipping 3082 + 3A > G	3rd	SD	2.5 mos

°updated in Caparica et al 2016

*pts died due to pneumonitis

§pt discontinued therapy due to pneumonitis

Ç concomitant MET amplification (MET/CEP7 ratio of 8)

Abbreviations: Smok. Status = smoking status; Hist. = histology; F-UP = follow-up; M = male; F= female; ADK = adenocarcinoma; Sq= squamous; CR = complete response; PR = partial response; mo(s) = month(s); NR = not reported

Tables S3- GEOMETRY-mono 1 and VISION phase II trials results

**Trial**	**GEOMETRY-MONO1 [74]**	**VISION [84-87]**
Drug	Capmatinib	Tepotinib
Pt N	364	337
**METex14**	151	152 (A)+ 161 (C)
1L/2L/≥2L	28+23/31/69	69+69/ 51/ 41+83
**ORR**	68%/48%/41%	62.3%/51%/ 44.6%
mDoR	12.6/6.9/9.7 mos	NE/12.6/ 11.1 mos
**mPFS**	12.4/8.11/5.4 mos	15.9/13.8/10.9 mos
**BM+**	13 (14 total)	15 pts
IC-ORR	7*/13 (54%): 4 CR	5/7 (70%)
**MET AMP**	84 (≥10 GCN)	24 (≥2.5 GCN on LB)
1L/2L/≥2L	15/3/66	7 /11/6
ORR	40%/0/29%	71%/27%/33%
mDoR	7.5/NE/8.3 mos	14.3/NE/NE
mPFS	4.2/2.8/4.1 mos	15.6/13.6/1.7

Abbreviations: Pt N = patient number; METex14 = MET exon 14 skipping mutations; MET AMP = MET gene amplification; GCN = gene copy number; LB = liquid biopsy; ORR = overall response rate; mDoR = median duration of response; mPFS median progression-free survival; BM+ = patients with brain metastases at baseline; IC-ORR = intracranial ORR; 1L = first-line; 2L = second-line; ≥2L = second-line or further; mos = months

Table S4 Ongoing clinical trials of MET inhibitors in patients with MET dysregulated NSCLC

Trial Identifier	Phase	Pt N	Drug(s)	Setting	Principal endpoint	BM permitted	comment
**Early stage**							
GEOMETRY-N NCT04926831	II	38	Perioperative capmatinib	Ib-IIIB	MPR	Not applicable	METex14 cohort 1 and GCN > 10 cohort 2
NCT05800340	II	30	Neo Toripalimab plus CT	IIb/IIIB	pCR	Not applicable	Neoadjuvant ICI in Rare Mutations NSCLC including METex14 or AMP
**MET-I**							
NCT05567055	II	35	capmatinib	Pts with BM	IC-ORR	Untreated asymptomatic BM must be > 1 measurable CNS lesion per RANO-BM	METex14 or MET AMP on plasma
NCT04739358	I/II	65	Tepotinib Tepotinib plus TKis	Pts with BM	IC-ORR	In the CNS efficacy cohort at least one measurable lesion must be intracranial	MET-driven NSCLC and BM or with MET driven resistance to other TKIs
NCT04566432	Obs	250	ICI or TTs	First-line	ctDNA mutation	asymptomatic BM or with stable symptoms	Therapeutic ResistAnce and Clonal Evolution Assessed With LB of NSCLC pts Treated With ICI or TTs
NCT04777175	Retrospective	186	ICI	Rare mutations	mPFS	No restrictions	Efficacy of ICI in pts with NSCLC harboring mutations including METex14 or AMP
MOMENT NCT05376891	Registry	700	All available therapies	NA	ORR/safety	No restrictions	Pts with METex14 NSCLC who are treated with a systemic therapy
MYLUNG NCT05885698	Consortium Obs.	7500	NA	NA	Genetic test rate	NA	All molecular drivers including MET
CROME NCT04084717	II	50	crizotinib	ROS-1 or MET+	ORR/PFS	Treated and asymptomatic BM are allowed	MET AMP or METex14
NCT05110196	IV	40	Capmatinib	MET-I naïve Indian pts	Safety	asymptomatic BM or with stable symptoms or previously treated with RT	METex14 skipping on tissue
GEOMETRY-C NCT04677595	II	35	Capmatinib	Naïve/MET-I naïve Chinese pts	ORR	asymptomatic BM or with stable symptoms	METex14 on tissue detectedby a central lab
NCT04777175	Obs	250	Capmatinib	Current indications in South korea	AEs	No restrictions	A Post Approval Commitment Study
TRUMP NCT03574402	II Umbrella	400	Several TKI	Gene-add- NSCLC	ORR	Asymptomatic BM Are allowed	Among 18 arms; METex14 crizotinib MET AMP ensartinib
NCT04322578	Obs	100	Crizotinib vs CT	Naive Chinese pts	PFS	NR	METex14 NSCLC analyzed for PFS and OS
NCT04575025	Obs	100	capmatinib	Current indication	AEs	All comers	All pts treated with capmatinib post-marketing period

NCT04923945	IV	163	Savolitinib	METex14	ORR	No restrictions	Cohort 1 previously treated with platin-based CT Cohort 2 treatment-naive
NCT01639508	II	86	Cabozantinib	MET driven	ORR	Previously treated BM and stable are allowed	Among the others a MET cohort is included
Novel drugs							
SPARTA NCT03175224	I/II	344	Bozitinib (APL-101; PLB1001)	MET-I naïve or refractory	MTD/ORR	asymptomatic BM or with stable symptoms	Solid tumors or NSCLC harbouring MET alterations
NCT04258033	II	185	Bozitinib (APL-101; PLB1001)	MET+	ORR	Asymptomatic and stable BM are allowed	Recruiting Chinese patients
NCT04992858	II	80	Ningetinib	MET-I naive	RP2D/ORR	asymptomatic BM or with stable symptoms or previously treated with RT	METex14 in plasma or tissue. **Prior therapies with MET-Is are not allowed**
SHIELD-1 NCT03993873	I/II	180	TPX-0022 (elzovantinib)	MET+ NSCLC, gastric or solid tumours	DLT/RP2D	Asymptomatic BM are allowed	METex14 or MET AMP No restrictions on prior MET-Is **Enrollment completed**
NCT04270591	I/II	183	Glumetinib (SCC244)	MET+/METex14	ORR	BM allowed only after definitive treatment	Cohort 1 (China) MET OE, MET AMP, METex14 Cohort 2 (USA) METex14 **Only in phase I Prior MET-Is allowed**
NCT02929290	Ib	80	BPI-9016M, a dual MET/Axl inhibitor	METex14 or MET OE	Safety and ORR	Asymptomatic BM stable and not requiring steroids for maintenance	Patients must have MET OE or METex14 **Prior type I MET-Is are allowed**
NCT04052971	I/II	78	ABN401 (selective MET-I)	MET+ NSCLC or solid tumours	Safety and ORR	BM allowed only after definitive treatment	Phase I MET+ Phase II METex14 **Only in phase I Prior MET-Is allowed**
NCT05752552	I	15	DO-2 (selective MET-I)	MET+ solid tumours	DLT/MTD	Asymptomatic and treated BM are allowed	MET mut or MET AMP (≥10 copies) **No restrictions on prior MET-Is**
CHRYSALIS NCT02609776	I	780	Amivantamab	EGFRmut/METex14	DLT/ORR	BM allowed only after definitive treatment	Cohort MET2: METex14 **Sub-cohort of pts pretreated with MET-Is**
NCT04868877	I/II	380	MCLA-129 (Anti-EGFR and Anti-c-MET Bispecific Ab)	METex14 naire or pretreated	MTD/ORR	asymptomatic BM or with stable symptoms or previously treated with RT	Solid tumors and or NSCLC **No restrictions on prior MET-Is**
NCT03797391	I/II	186	EMB-01 (Anti-EGFR and Anti-c-MET Bispecific Ab)	Solid tumours	MTD/ORR	Asymptomatic BM are allowed	Phase I solid tumours Phase II EGFR+ or MET+ NSCLC **No restrictions on prior MET-Is**
NCT04667975	I/II	74	CKD-702 (Anti-EGFR and Anti-c-MET Bispecific Ab)	NSCLC	MTD/ORR	No restrictions	Unselected **No restrictions on prior MET-Is**
NCT05332574	I/II	120	GB263T (Anti-EGFR/MET tri-specific Ab)	NSCLC or solid tumours previously treated	DLT/ORR	BM allowed only after definitive treatment	Solid tumors and or NSCLC **No restrictions on prior MET-Is**

Sym015.01 NCT02648724	I/II	57	Sym015 (mixture of MET abs)	Solid tumours	DLT/ORR	BM allowed only after definitive treatment	Exp cohort for MET AMP or METex14 **No restrictions on prior MET-Is Enrollment completed**
NCT04077099	I/II	82	REGN5093	MET+ NSCLC	Safety/ORR	BM allowed only after definitive treatment	METex14, MET AMO or MET OE. **Only in phase I Prior MET-Is allowed Enrollment completed**
NCT05029882	I	300	ABBV-400 Teliso-Top1-I (ADC)	Solid tumours	ORR	No restrictions	MET OE EGFRWT or EGFR+ or squamous NSCLC **MET-Is are not standard treatment for this pt population**
Luminosity NCT03539536	II	275	Teliso-Vedotin (ADC)	MET OE previously treated	ORR	BM allowed only after definitive treatment	c-Met+ as assessed by an AbbVie designated IHC laboratory **MET-Is are not standard treatment for this pt population**
NCT05513703	II	70	Teliso-V (ADC)	**Tx naive** MET AMP	ORR	BM allowed only after definitive tretament	MET AMP detected on tissue or liquid biopsy by Sponsor approved test **Prior MET.I not allowed due to study design**
TeliMET Lung01 NCT04928846	III	698	Teliso-V vs docetaxel	MET OE	PFS/OS	BM allowed only after definitive tretament	EGFR WT and MET OE (by an AbbVie designated laboratory) **Prior MET.I not allowed due to study design**
NCT04982224	I/II	83	REGN5093-M114 (ADC)	MET OE solid tumors	Safety	Treated and stable BM are allowed	MET OE **MET-Is are not standard treatment for this pt population**
NCT05652868	I	150	MYTX-011 (ADC)	NSCLC	DLT/ORR	Treated and stable BM are allowed	Part 1 unselected Part 2 MET OE confirmed by central laboratory testing **MET-Is are not standard treatment for this pt population**
Combo trials							
METalmark NCT05488314	Ib/II	161	Capmatinib plus amivantamab	Naive or previously treated	RP2D/ORR	asymptomatic BM or with stable symptoms	METex14 or AMP
NCT05435846	Ib	33	Capmatinib plus trametinib	MET-I refractory	RP2D/safety	previously treated BM with RT and stable	METex14 on plasma or tissue. Note MET-I is not mandatory to be the last line of treatment
POTENT NCT05782361	Ib	38	Tepotinib plus pembrolizumab	Naïve or ICI refractory	ORR iRECIST	asymptomatic BM or with stable symptoms or	METex14 on plasma or tissue

						previously treated with RT	
SOUND NCT05374603	II	60	Savolitinib plus durvalumab	MET+	PFS	Asymptomatic BM are allowed	MET OE, MET AMP or METex14
NCT05777278	I/II	29	Savolitinib plus docetaxel	2L MET OE	ORR	Asymptomatic BM, stable, are allowed	MET OE (3+ in ≥50% of TC)

Abbreviations = pt(s) N = patient(s) number; BM = brain metastases; MPR = major pathological response; pCR = pathological complete response; MET OE = MET overexpression; METex14 = MET exon 14 skipping mutations; MET AMP = MET amplification; GCN = Gene copy number; TC = tumor cells; (IC)- ORR = (intracranial)- overall response rate; iRECIST = immune-response evaluation criteria in solid tumors; mPFS = median progression-free survival; OS = overall survival; MTD = maximum tolerated dose; DLT = dose limiting toxicity; RP2D = recommend phase 2 dose; Tx Naïve= treatment naïve; 2L = second-line; CT= chemotherapy; RT = radiotherapy; ICI = immune checkpoint inhibitors; TKIs = tyrosine kinase inhibitors; TT(s) = targeted therapy; MET-I(s) = MET inhibitor(s); ADC= antibody drug conjugated; ctDNA = circulating DNA; NA = not applicable; obs. = observational; gene-add. = gene addicted; NSCLC = Non-small-cell lung cancer; AEs = adverse event(s);

Table S5- Clinical trials of type II MET inhibitors in patients with MET dysregulated NSCLC

**Trial identifier**	**Drug**	**Phase**	**Pt N**	**Targeted pt population**
NCT02132598	Cabozantinib	II	5	Previously treated pts with BM and MET AMP (ratio ≥ 2), Trial stopped due to slow patient accrual.
CABinMET NCT03911193	Cabozantinib	II	25	Previously treated pts with METex14 or MET AMP (ratio ≥ 2.2). Prior treatment with MET-I is allowed
NCT01639508	Cabozantinib	II	86	Group D is catered for MET OE, AMP or Mut. No restrictions on prior TKIs
Kollmannsberger 2023 [157] NCT00697632	Glesatinib	I	179	Trial enrolled pts with OE, AMP or METex14 NSCLC. There were 6 PRs in METex14
NCT02544633	Glesatinib	II	68	MET dysregulated NSCLC. No prior MET-Is
NCT02920996	Merestinib	II	12	METex14 or NTRK1,2,3 + NSCLC. No restrictions on MET-Is

Abbreviations: Pt N = patient number; pt(s) = patient(s); BM= brian metastases; MET AMP = MET amplification; METex14 = MET exon 14 skipping mutations; MET-I(s) MET inhibitor(s); MET OE = MET overexpression; Mut = mutation; TKIs = tyrosine kinase inhibitors; NTRK = Neurotrophic tyrosine receptor kinase ; NSCLC = Non-Small-Cell lung Cancer
